# Clinical considerations for immune dysregulation and immunodeficiency in Down syndrome

**DOI:** 10.70962/jhi.20260052

**Published:** 2026-07-15

**Authors:** Melissa Gans, Juanita Valdes Camacho, Matthew Wyke, Kasama Manothummetha, Harry Lesmana, Jacqueline Squire, Joao Pedro Lopes, Erica G. Schmitt, Hanadys Ale, Junghee J. Shin, Natalia Chaimowitz, Carolyn Baloh, Dusan Bogunovic, Joaquin Espinosa

**Affiliations:** 1 Jackson Health System, University of Miami Miller School of Medicine, Miami, FL, USA; 2 LSU Health Shreveport, Shreveport, LA, USA; 3 Cleveland Clinic, Cleveland, OH, USA; 4 Mayo Clinic Jacksonville, Jacksonville, FL, USA; 5 UH Rainbow Babies and Children’s Hospital, Case Western Reserve University School of Medicine, Cleveland, OH, USA; 6 Washington University in St. Louis School of Medicine, St. Louis, MO, USA; 7 Memorial Healthcare System, Hollywood, FL, USA; 8 Yale University, New Haven, CT, USA; 9 Cook Children’s Medical Center, Fort Worth, TX, USA; 10 Brigham and Women’s Hospital, Boston, MA, USA; 11 Columbia University Medical Center, New York, NY, USA; 12 Linda Crnic Institute for Down Syndrome, University of Colorado Anschutz, Aurora, CO, USA

## Abstract

Down syndrome (DS), the genetic condition caused by trisomy 21 (T21), is characterized by lifelong immune dysregulation leading to high rates of autoimmune disorders, elevated risk of complications from infections, immune hypersensitivity, and a unique form of immunodeficiency. It is now appreciated that DS shares key hallmarks with interferonopathies, with vast remodeling of all branches of the immune system, hypercytokinemia, and widespread autoantibody production. Here within, we review the existing literature with an emphasis on clinical considerations toward monitoring, management, and therapeutic opportunities. We highlight recent research advances that illuminate diagnostic approaches to evaluate immune dysregulation in DS. We also discuss the evidence supporting specific immunomodulatory strategies that could have multidimensional benefits in this population, including JAK inhibitors, intravenous immunoglobulin, and B cell–depleting agents.

## Teaser

Down syndrome (DS) is characterized by lifelong immune dysregulation leading to high rates of autoimmune disorders, complications from infections, immune hypersensitivity, and a unique form of immunodeficiency. Here within, we describe clinical considerations toward monitoring, management, and therapeutic opportunities. We highlight recent research advances that illuminate diagnostic approaches to evaluate immune dysregulation in DS.

## Introduction

**Figure 1.**
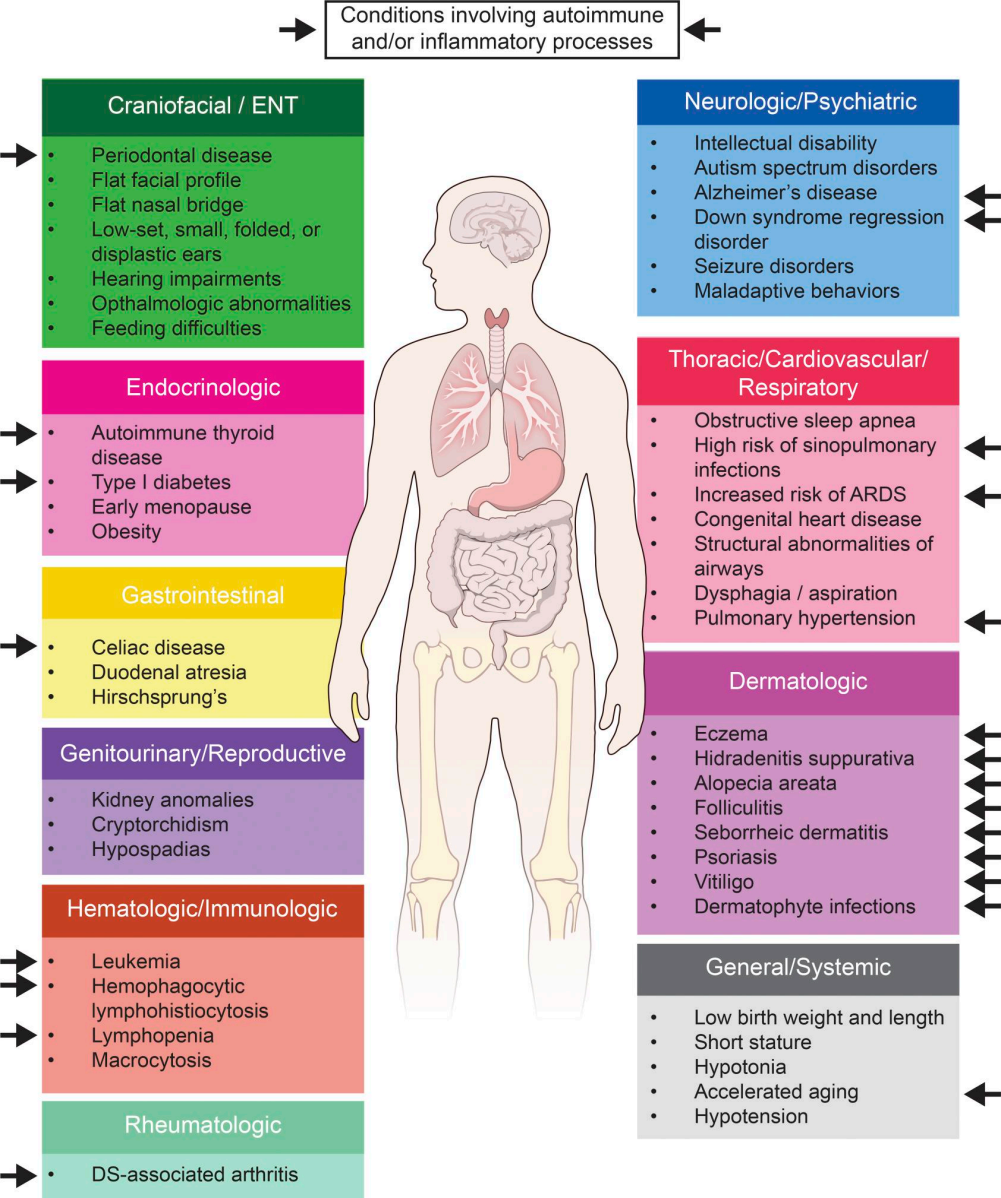
**Overview of clinical manifestations and physical exam findings in patients with D** **S** **, highlighting those involving autoimmune and/or inflammatory processes.**

Down syndrome (DS), or trisomy 21 (T21), is the most prevalent chromosomal abnormality and a leading cause of intellectual and developmental disability worldwide (1). Thanks to advancements in social inclusivity and specialized medical care, individuals with DS now enjoy extended lifespans into adulthood (2). DS is characterized by varying degrees of cognitive impairments, delayed growth, and organ dysmorphogenesis, and increased risk of a wide range of co-occurring conditions affecting every major organ system, many of which involve autoimmune and/or inflammatory processes (Fig. 1) (1). T21 causes a differential clinical risk profile, including higher rates of congenital heart defects (CHDs), hearing and vision impairments, hypotonia, obstructive sleep apnea, hematologic abnormalities, and neurological conditions such as autism spectrum disorder and seizure disorders (3). People with DS also display increased risk of various autoimmune diseases (4, 5), though remarkably, they show lower rates of most solid tumors (6), hypertension, and myocardial infarction (7).

Individuals with DS exhibit increased risk of complications from infections, which are a leading cause of mortality and increased medical burden in this population (8). In particular, individuals with DS are disproportionately susceptible to recurrent sinopulmonary infections (9). Historically, this vulnerability has been attributed to non-immunological contributors such as structural abnormalities of the airways, obstructive sleep apnea, congenital malformations within the lower respiratory tract, and congenital heart disease (10). These conditions can compromise respiratory function and create an environment more conducive to infections (11, 12). However, we now know that there is also a confluence of factors impacting their immune defense mechanisms, leading to an increased awareness of the immunodeficiency and immune dysregulation of DS. While there is not sufficient data to conclude that patients with DS have a higher rate of infection, there are clearly more severe consequences from infections in DS, and this defines what we are considering the immunodeficiency of DS. Furthermore, the well-established increased risk of autoimmunity in DS and the inability of the immune system in DS to properly regulate its responses to stimuli (13, 14, 15, 16) lead to a state of chronic immune dysregulation in DS.

Approximately 1 in 700 babies are born with DS each year in the U.S. alone (17). Despite being the most common genetic syndrome associated with immune defects (18), DS is not currently included in the 2024 classification of inborn errors of immunity compiled by the International Union of Immunological Societies (IUIS) Expert Committee (19). Furthermore, the current health supervision guidelines for children and adults with DS do not directly address the unique aspects of the immune system in this population and do not provide any specific recommendations for immune evaluation or surveillance (3, 20).

In recent years, there has been a growing body of literature describing the pathophysiology of immune dysfunction in DS that could explain why these individuals are more prone to complications from infections and immune disorders. This article reviews the latest knowledge on immunity in DS with a focus on practical clinical considerations for routine screening of immune dysfunction in this population, along with potential treatment options. This work came out of an effort by the Members of the Clinical Immunology Society Early Career Immunologist Committee and Members of the Down Syndrome Medical Interest Group – USA Immune Disorders Working Group.

There are several excellent reviews that cover the latest research developments in the study of molecular and cellular mechanisms of immune dysregulation in DS (5, 21, 22). We focus here on key areas of clinical relevance in terms of approaches for evaluation of immune disorders in DS and potential therapeutic approaches.

## Autoimmunity in DS

Individuals with DS have higher rates of autoimmune conditions compared to age- and sex-matched control populations (4, 23) (Fig. 1). Autoimmune thyroid disease (AITD) is highly prevalent in DS, with >50% of adults eventually developing thyroid dysfunction, mostly in the form of hypothyroidism (24). The American Academy of Pediatrics and the Global Down Syndrome Foundation Medical Care Guidelines for Adults with DS underscore the importance of screening for AITD (3, 20), as early detection and prompt treatment are critical. In a recent study of >200 individuals with DS, >50% of adults tested positive for antithyroid peroxidase (TPO) autoantibodies (25), just one of the many autoantibodies associated with AITD. After AITD, autoimmune skin conditions are the second most prevalent group of autoimmune disorders in DS—including but not limited to alopecia areata, psoriasis, and vitiligo (26). Atopic dermatitis, seborrheic dermatitis, folliculitis, hidradenitis suppurativa, and many other immunodermatological conditions are also quite common in DS. Individuals with DS are also at increased risk of celiac disease, which is ∼10-fold more common in this population (27). Celiac disease is underdiagnosed in DS (28) and characterized by lower frequency of high-risk HLA alleles (29, *Preprint*). In those patients with celiac disease and without DS, over 99% have a high-risk HLA allele. However, in those patients with celiac disease and with DS, the frequency of high risk HLA alleles is lower (29, *Preprint*), suggesting the existence of additional factors contributing to celiac disease pathogenesis in DS. Other autoimmune disorders more common in DS include type 1 diabetes (T1D) (3, 20) and a form of arthritis referred to as arthropathy of DS or DS-associated arthritis (30). T1D shows unique features in DS, including an earlier age of onset (often before age 5), a differential pattern of autoantibodies, and lower rates of typical T1D-associated HLA alleles (31). DS-associated arthritis also has a distinctive presentation, typically appearing as polyarticular-rheumatoid factor-negative arthritis, with predominance in the small joints of the hands and wrists (32). Furthermore, DS-associated arthritis can be distinguished from juvenile idiopathic arthritis by dysregulation of follicular helper T, peripheral helper T, and regulatory T cells (Tregs) (33). Notably, one study found that ∼80% of immune disorders are diagnosed during childhood in DS, with minimal sex bias, and with only slightly higher rates of AITD and hidradenitis suppurativa in females (25).

Recent reports demonstrate high rates of autoantibody production targeting all major organ systems in DS (25, 34), indicating that many other clinical hallmarks could have an autoimmune basis in this population. For example, Down syndrome regression disorder (DSRD), a condition marked by acute neurocognitive decline (35), has been associated with immunoglobulin synthesis and hypercytokinemia in the central nervous system (36).

While the exact underlying mechanisms driving autoimmunity in DS are yet to be fully elucidated, multiple mechanisms could be at play, most prominently hypercytokinemia and dysregulation of the T and B cell lineages. Overexpression of chromosome 21 genes with potent immune functions is likely to contribute to this phenomenon, including genes encoding four interferon (IFN) receptor subunits (*IFNAR1*, *IFNAR2*, *IFNGR2*, and *IL10RB*) (37) and the autoimmune regulator (*AIRE*) transcription factor (38). Elevated baseline activation of IFN signaling and downstream JAK/STAT signaling is well established in DS (13, 14). Hyperactive IFN signaling is a known risk factor for autoimmunity in the general population, as demonstrated by genetic variants leading to increased activity in this pathway (39) and by the known high risk of autoimmunity upon therapeutic administration of IFNs (40). In a large multi-omic study of the population with DS, IFN scores were positively associated with both the degree of hypercytokinemia and the extent of T and B cell remodeling (41). We can hypothesize that hypercytokinemia is a downstream effect of this increased IFN activation as IFN-stimulated T cells with T21 overproduce pro-inflammatory cytokines (13). As for the triplication of AIRE, AIRE expression is paradoxically decreased in DS thymuses (42, 43). This AIRE dysregulation causing a partial central failure likely allows autoreactive T cells to escape the thymus and, in the setting of an interferonopathy, contribute to the hypercytokinemia observed in DS.

Beyond elevated IFN responses, key cytokines elevated in DS that are known drivers of autoimmunity in the general population include tumor necrosis factor (TNF)α, IL-6, IL22, CXCL10, CCL2 (MCP1), and various IL17 isoforms (25, 44, 45). This inflammatory milieu can dysregulate T and B cells toward an autoimmunity-prone state, including shifts in T cell subsets toward effector, memory, and exhausted types refractory to Treg-mediated suppression (13, 14), as well as dysregulation of B cell subsets toward autoreactive states, such as and increased frequency of CD11c^+^Tbet^+^CD21^low^ B cells (14, 34), which are associated with autoantibody production in the typical population.

### Evaluation of autoimmunity in DS

Autoantibody production is widespread in the population with DS and of uncertain relevance in terms of affecting clinical management. An analysis of 25 autoantibodies elevated in DS found that almost 100% of research participants were positive for at least one of these (25). Key autoantibodies that could influence clinical practice in individuals with DS are those associated with AITD (anti-TPO, anti-thyroglobulin, antithyroid-stimulating hormone receptor), celiac disease (e.g., anti-tissue transglutaminase, antideamidated gliadin peptide), and T1D autoantibody panels. People with DS also show high rates of positivity (over 60%) for antinuclear antibodies (25). The presence of autoantibodies needs to be interpreted cautiously by clinicians in light of concurrent symptoms and overall clinical presentation. New onset of a co-occurring condition may elicit testing for autoantibodies associated with similar symptomology in the general population. For example, anti-MUSK (muscle specific kinase) autoantibodies, which underlie some forms of myasthenia gravis, were found to be elevated in DS (25) and may inform the etiology of similar neuromuscular symptomology in persons with DS.

People with DS also display hypocomplementia, with strong depletion of circulating levels of multiple complement subunits (45). This may be indicative of sustained complement activation and consumption downstream of autoantibody reactivity, as seen in systemic lupus erythematosus (SLE). High autoantibody levels concurrent with pronounced hypocomplementia could indicate a stronger autoimmune phenotype in DS.

## Immune dysregulation in DS

### Dysregulation of innate immunity in DS

**Figure 2.**
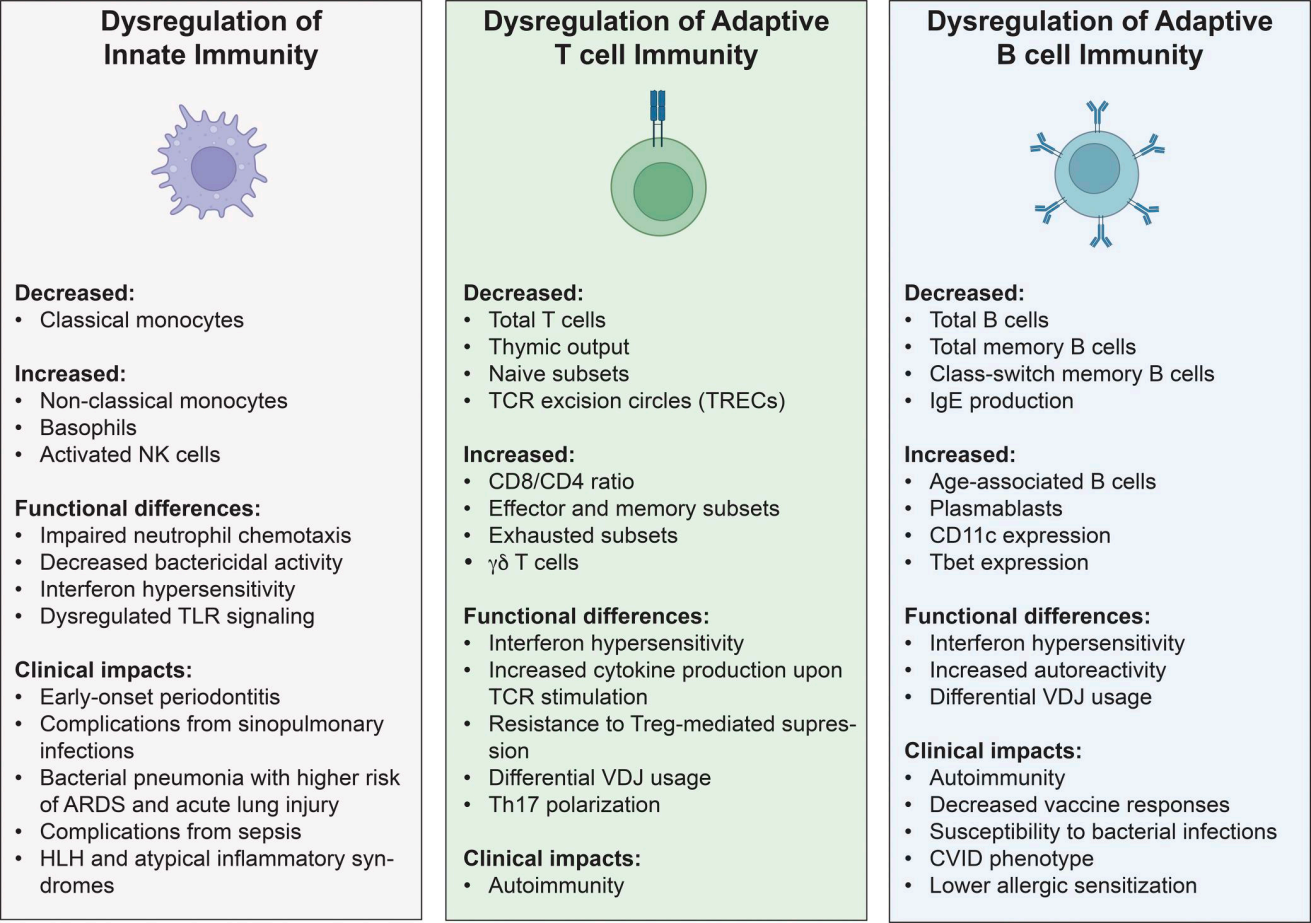
Mechanisms and clinical manifestations of dysregulated innate and adaptive immunity in DS.

In DS, dysregulation of innate immunity may contribute to both increased complications from infections and autoinflammation (Fig. 2).

Whereas some studies have demonstrated decreased neutrophil counts in young children with DS (46, 47), this has not been observed consistently in larger cohorts, including broader age ranges (41, 48). Expectedly, decreased overall leukocyte count was associated with infection-related hospitalizations in DS (47). Impaired neutrophil chemotaxis and bactericidal capabilities have been described in DS, yet phagocytosis and oxidative burst do not appear to differ significantly (49). Intrinsic dysregulation of neutrophil intracellular calcium concentrations, important for cellular function, has also been documented (50). Clinically, the effects of dysregulation of neutrophils in DS may contribute to increased rates of bacterial infections in these patients. Neutrophils are crucial for preventing sepsis (51), and neutrophil dysfunction in DS likely contributes to this increased septicemia-related mortality in this population (52, 53). Furthermore, patients with DS are at high risk for early-onset periodontitis (54) for unclear reasons, but we can hypothesize that DS neutrophil dysfunction likely contributes to this phenotype based on the fact that patients with known neutrophil disorders such as leukocyte adhesion deficiency have periodontal disease (55).

Similar to neutrophils in DS, monocytes show impaired phagocytosis and chemotaxis (56), yet they maintain bactericidal function and several components of the IFN pathway are upregulated, which has important implications for viral protection and autoinflammation (5, 16, 57). Clinically, monocytes, particularly macrophages, are believed to be important in acute respiratory distress syndrome (ARDS) (58). Patients with DS are at high risk for acute lung injury and ARDS from bacterial pneumonia and impaired monocyte function may contribute to this clinical finding (59).

Basophils counts are elevated in DS, concurrent with lower eosinophil counts, indicative of myeloid skewing (34, 41), but these differences mostly resolve in adulthood (48). The impact of basophilia in DS is not known. However, as described later, people with DS show much lower levels of IgE (45), which would impair IgE-mediated responses via basophils and may explain the lower rate of allergic sensitization in this population (60).

Natural killer (NK) cell abnormalities via alterations in NK cell numbers or function have been documented in DS (61, 62, 63, 64). Studies have shown an increased frequency of a cytotoxic NK cell population (CD16^+^CD56^+^ NK cells) that express increased markers of NK cell cytotoxicity and activation (14). This NK cell dysfunction likely contributes to the hemophagocytic lymphohistiocytosis (HLH) and atypical inflammatory syndrome seen in some individuals with DS (64, 65). Lastly, there is also evidence of immune dysregulation involving toll-like receptor pathways in DS patients (66).

### Evaluation of innate immunity in DS

In clinical practice, annual profiling of peripheral blood counts with differential to screen for neutropenia alongside flow cytometry for lymphocyte phenotyping may help stratify DS patients at risk for more significant infections and immune dysregulation. Clinicians should also be aware of potential abnormalities in monocytes, eosinophils, and basophils that may be seen on this differential and are typical in DS. However, even with normal total cell counts, there are likely abnormalities at a functional level that would require more detailed phenotyping not readily available commercially. Additionally, lymphocyte phenotyping should be interpreted by an immunologist able to discern age-adjusted normal values from lymphopenia. Furthermore, in clinical practice, patients with DS also should have early and frequent screening for periodontal disease (54) and early, aggressive treatment of septicemia (53).

### Abnormalities in T cell–driven adaptive immunity

In DS, an abnormality in the thymic compartment associates with disruption of central tolerance, leading to higher susceptibility to infections and autoimmune disease (67, 68) (Fig. 2). In individuals with DS, the thymus is typically smaller and contains fewer cells—most noticeable in infants with DS, with reduced thymic output (69), and reduced thymic emigrants (70). There is also an impact on thymic function with diminished proportion of mature thymocytes, as defective T cell maturation is an early feature of DS (71). The thymus in people with DS also shows an overexpansion of γ/δ T cells compared to age-matched controls (72). Studies have shown changes in the thymic stroma in DS that include fibrotic involution concerning both cortical and medullary microenvironments (73), along with defects in both immature and mature lymphocytes, likely contributing to the autoimmune disorders observed in this population (74, 75). A key indicator of this thymic underdevelopment is the reduced number of T cell receptor excision circles (TRECs) found in the peripheral blood of DS children, compared to euploid counterparts (76). Low TRECs are used in newborn screen programs to identify babies with severe combined immunodeficiency (SCID) (77). This assay also detects infants with non-SCID T cell lymphopenia, as can occur in DS patients. Further, there is a decrease in the overall count of peripheral T cells and an imbalance in their subsets (9, 78). Specifically, there is an increased CD8^+^/CD4^+^ ratio along with decline in the naive subsets of CD4^+^ and CD8^+^ T cells, leading to an increased proportion of effector and memory T subsets (13, 14, 79). The function of the T cells is also impaired in DS with reduced lymphocyte proliferation to phytohemagglutinin (80), which tends to worsen over time in DS (81). Lymphocytes in DS show signs of premature aging (82) due to unknown reasons that are likely global in DS, contributing to other aspects of the DS phenotype in addition to T cell exhaustion (83). Additionally, reduced inhibitory function of Treg cells has also been reported (79, 84). The frequency of Tregs among T cells is actually higher in DS, though functional assays show that effector T cells in DS are resistant to Treg-mediated suppression (13). Mechanistically, this phenomenology is accompanied by increased baseline JAK/STAT signaling, as evidence by elevated levels of phosphorylated STAT transcription factors (similar to that seen in STAT3 gain-of-function syndrome) (13, 14, 37), that can be counteracted with JAK inhibition or IL-6 blockade (34).

### Abnormalities in B cell–mediated adaptive immunity

From an immunological standpoint, defective B cell–mediated humoral responses are likely to contribute to the increased susceptibility to the high risk of autoimmunity and complications from sinopulmonary infections in DS patients (Fig. 2). Studies investigating serum immunoglobulin levels have yielded conflicting results, with reports of normal, decreased, or even elevated levels (47, 74, 81, 85, 86, 87, 88). This is likely due to the various phenotypes of DS, as there is a distinct subset of DS patients with hypogammaglobulinemia who fit a common variable immunodeficiency (CVID) diagnosis yet others with normal immunoglobulins and specific antibody responses (9, 89). However, even in the absence of hypogammaglobulinemia, research suggests a decline in class switch memory B cells and a diminished specific antibody response in DS patients compared to controls (90, 91, 92, 93, 94, 95, 96, 97, 98). This indicates an underlying defect in B cell development and function, despite potentially normal immunoglobulin levels. Interestingly, persons with DS typically have low IgE (45) and are less likely to have aeroallergen sensitization (99). However, interestingly and with unknown mechanism, there is evidence to suggest that patients with DS are at higher risk for non-IgE-mediated food allergies and more severe reactions to non-IgE-mediated food allergies (100, 101).

Lower B cell counts are observed across the lifespan in DS and severe B cell lymphopenia is a frequent finding in adults with DS, suggesting an age-related exacerbation of the B cell deficiency (102). B cell profiling in ∼300 individuals with DS versus ∼100 euploid controls across the lifespan demonstrated that B cell dysregulation occurs in distinct phases, with progressive increases in age-associated B cells (ABCs) and plasmablasts (48). High proportion of plasmablasts and CD11c^+^Tbet^high^CD21^low^ B cells is likely to indicate higher frequencies of autoreactive B cells (34).

### Evaluation of adaptive immunity in DS

We recommend that all patients with DS should be evaluated for T cell lymphopenia by flow cytometry for CD3, as well as CD4 and CD8 subtypes. In practice, functional studies of T cell function are likely not clinically useful for patients with DS but can be considered in the subset of individuals who are profoundly lymphopenic. Furthermore, as graft-versus-host disease or T cell receptor oligoclonality has not been described in lymphopenic patients with DS, unlike in patients with SCID or DiGeorge syndrome, evaluation of naive and memory T cell subsets is unlikely to be clinically meaningful in this population.

These findings also underscore the importance of a comprehensive evaluation of B cells, both quantitatively and qualitatively, in individuals with DS. These patients should, at a minimum, have quantification of CD19 cells by flow cytometry along with IgG, IgA, IgM, and IgE levels. Measuring IgG subclasses is unlikely to be clinically useful in DS, though subclass deficiency does exist in DS (103). While B cell phenotyping may be abnormal in patients with DS, it is not widely commercially available and is unlikely to be clinically useful or change management in practice. Humoral responses should be measured by looking at specific vaccine titers, most notably to *Streptococcus pneumoniae* (104) and some protein antigen titers. Patients with DS and poor vaccine titers can benefit from repeat vaccination. The authors consider that all patients with DS could benefit from booster pneumococcal administration after the age of 2, as DS is an immunocompromising condition and patients with DS are known to have poor responses to pneumococcal vaccination (97, 98, 104). Furthermore, vaccination against common respiratory viruses such as respiratory syncytial virus (RSV), influenza, and COVID is of utmost importance for DS, as these patients are at high risk of RSV lower respiratory tract infections (105) in addition to high risk of mortality from COVID19 (106, 107) and influenza (108).

All patients with DS should be evaluated by an immunologist to determine if they meet criteria for CVID or specific antibody deficiency (SAD) and if so, with a history of recurrent sinopulmonary infections, immunologists should have a low threshold to initiate immunoglobulin replacement therapy and/or prophylactic antibiotics. Patients with DS should be evaluated for CVID and SAD, and if meet guidelines for CVID or SAD diagnosis by immunological testing and history of recurrent or severe infections, they should be treated with replacement immunoglobulin at standard doses of 400–600 mg/kg/month (109, 110). Prophylactic antibiotics can also be considered but immunoglobulin will be more effective. However, in patients with DS and immunological abnormalities not profound enough to meet diagnostic criteria for CVID or SAD, patients should still be considered for replacement immunoglobulin if recurrent or severe infections. We know that there is always some degree of immunodeficiency and immune dysregulation in DS that may not translate to commercially available standard immunological testing. Thus, while immunoglobulin cannot completely correct the underlying immune defect, it can help prevent severe and recurrent bacterial and viral infections in this vulnerable population.

## Interferonopathy in DS

**Figure 3.**
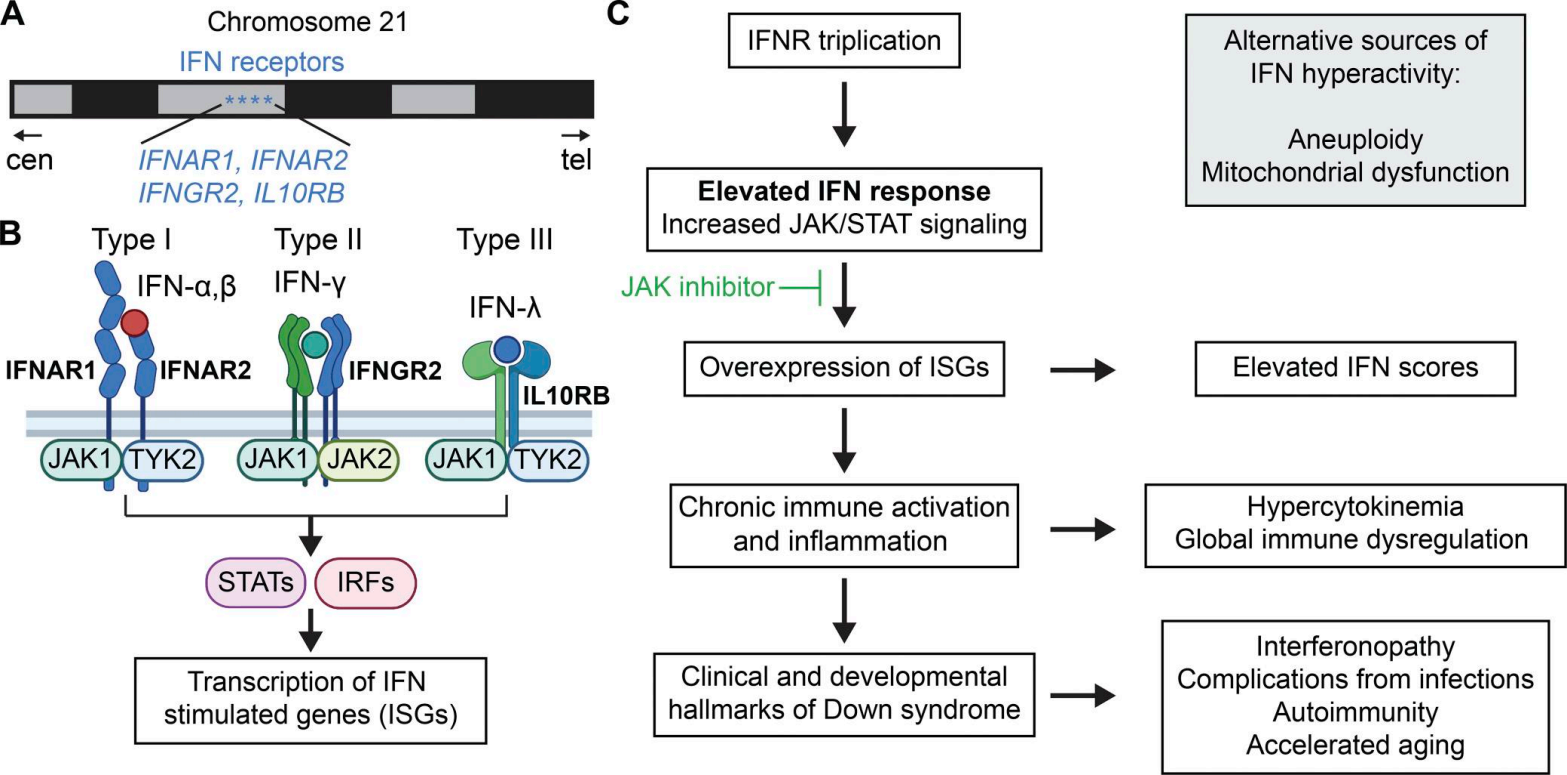
**The interferonopathy of DS.** **(A)** Panel shows the location of IFN receptors on chromosome 21. **(B)** Panel illustrates the types of interferons and their respective receptors interacting with JAK1 and TYK2 proteins. **(C)** Panel depicts pathophysiology causing the interferonopathy in Down syndrome.

It is now accepted that DS shares pathophysiological processes with interferonopathies (13, 22, 57, 111) (Fig. 3). DS causes consistent baseline elevation of the IFN transcriptional response across immune and nonimmune cell types, along with hypersensitivity to IFN ligands and over-induction of downstream JAK/STAT signaling (13, 14, 57, 112). The cytokinopathy of DS includes elevation of many known IFN-inducible cytokines, chemokines, and interleukins, including but not limited to CXCL9, CXCL10 (IP-10), CXCL11, CCL2 (MCP-1), IL6, IL12, and TNFα (34, 45). Additionally, T21 activates the IFN-inducible kynurenine pathway, leading to the production of neurotoxic tryptophan catabolites, such as quinolinic acid, via induction of IDO-1, the rate-limiting enzyme in the pathway (113). Mechanistically, these observations can be explained in part by the triplication of the four IFNRs encoded on chromosome 21 (37, 57). In a murine model of DS, interferon production regulator (IFNR) triplication drives multiple phenotypes of DS, including lethal hypersensitivity to viral mimetics, CHDs, developmental delays, cognitive impairments, and craniofacial malformations (37).

Noteworthy, the interferonopathy of DS has many differential features relative to other IFN-driven disorders. First, it is a mixed type interferonopathy involving all three types of IFN signaling. In the bloodstream, IFN scores track strongly with baseline levels of IFNγ (type II) and IFNλ (type III), but in other tissues, the type I IFN response may be more dominant (41). This mixed type may explain phenotypic commonalities and differences relative to monogenic type I interferonopathies. This could have implications from a management standpoint, as JAK inhibitors with different target specificity are increasingly available (15, 22, 111). Second, the interferonopathy of DS is associated with IFNR overexpression and not with IFN ligand overproduction as seen in monogenic type I interferonopathies (41). This has clinical implications because the interferonopathy of DS is more accurately monitored through transcriptional scores capturing elevation of IFN-stimulated genes (ISGs) and plasma levels of IFN-inducible factors (e.g., CXCL10, B2M) and not necessarily by measuring levels of the IFN ligands themselves (41). Third, because the interferonopathy of DS is likely driven by IFNR overexpression, it manifests as a mild interferonopathy in the absence of immune triggers, but it can be strongly exacerbated by IFN-inducing immune stimuli (15, 37, 57). For instance, this heightened inflammatory response is noted with severe atypical inflammatory phenotypes triggered by SARS-CoV-2 (65, 114), RSV (115), and other viruses (116) in DS. It has also been documented in hyper-inflammatory responses that fit HLH criteria in patients with DS (64).

In summary, IFN hyperactivity could be a major driver of immune phenotypes in DS, with both common and unique features relative to other IFN-driven conditions.

### Evaluation of interferonopathy in DS

Broad-based screening for the interferonopathy in DS is currently difficult, as there is limited commercial availability of advanced laboratory tests that evaluate interferonopathies. As mentioned earlier, the interferonopathy of DS is better assessed by evaluation of downstream signaling events, such as elevated expression of ISGs through RNA-based IFN scores or measurements of plasma levels of IFN-inducible factors, instead of measurements of IFN ligands.

In the Human Trisome Project, a large cohort study of people with DS (NCT02864108), RNA-based IFN scores derived from a subset of 18 ISGs measured via whole blood transcriptome were found to correlate positively with the degree of peripheral immune remodeling, cytokine elevation, activation of the kynurenine pathway of tryptophan catabolism, and history of AITD and CHD in DS (13, 14, 37, 41, 113). On average, these IFN scores were >10-fold higher in DS relative to healthy euploid controls, with an elevation on par with the degree of IFN hyperactivity observed upon SARS-Co-V2 infections in the general population (41). IFN scores show strong inter-individual variability in DS, but do not change significantly by age or sex (41). Importantly, IFN scores were decreased to within normal ranges upon JAK1/3 inhibition with tofacitinib (25, 41). Commercially available IFN score tests include the type I IFN test using four ISGs developed by DxTerity, which has been employed extensively in SLE (117). Of the four ISGs measured in this test (HERC5, IFI27, IFIT1, and RSAD2), only two of them (IFI27 and RSAD2) are significantly elevated in the whole blood transcriptome of people with DS, bringing into question whether this test would perform well in DS (41). Commercial development of other RNA-based IFN scores is already underway, showing great promise in monitoring patients with SLE at risk for progression to lupus nephritis (Rovin, B., et al. 2019. ACR/ARP Annual Meeting. https://acrabstracts.org/abstract/increased-risk-of-progression-to-lupus-nephritis-for-lupus-patients-with-elevated-interferon-signature/). A customized IFN score specific for DS may have to be developed and tested for broader use in this population.

Many soluble factors in plasma or serum could be used as surrogates for IFN hyperactivity in DS, including CXCL10, CXCL11, CXCL9, GZMA, and B2M. All of these factors are elevated in DS, correlate positively with RNA-based IFN scores (41), and can be measured with commercially available tests through several labs in the United States.

Metabolic markers associated with the interferonopathy of DS include kynurenine and its derivative quinolinic acid, which are often included in metabolic panels measuring a wide range of amino acids and their byproducts. Elevated levels of neopterin and ferritin can also be proxies for elevated IFN signaling, mostly IFNγ. However, hyperferritinemia has not been studied specifically in DS patients (118, 119).

The authors recommend that providers caring for persons with DS should be aware of the potential interferonopathy in these patients. This can clinically manifest by unexplained fevers, critically ill with unknown cause, or signs of systemic inflammation such as weight loss and fatigue. When immune dysregulation and inflammation are recognized in this vulnerable population, the authors recommend considering prompt and aggressive treatment with immunomodulators.

## Therapeutic implications

### JAK inhibitors in DS

Chronic activation of IFN signaling is a defining feature of DS, driven in part by triplication of type I, II, and III *IFNR* genes encoded on chromosome 21 (Fig. 3). This gene-dose effect enhances JAK-STAT signaling and lowers the threshold for inflammatory activation across multiple immune and nonimmune cell lineages (14, 15, 57, 112). Preclinical modeling in mouse models of DS reinforces this mechanism (15, 41, 120, *Preprint*), and human studies provide convergent evidence (14, 57). Given that all three types of IFN signaling employ JAK kinases for signal transduction, JAK inhibitors could provide therapeutic benefit in DS (112). In fact, an unbiased genetic screen of the human kinome identified members of the JAK family as the protein kinases with the most detrimental effects on the viability of cells with T21 (112). Given that all three types of IFN signaling employ JAK1 for signaling transduction in combination with TYK2 or JAK2, it is expected that JAK1 inhibitors may be more effective at controlling heightened IFN responses in DS.

Early case reports documented beneficial effects of JAK inhibition for the treatment of alopecia areata (121), psoriatic arthritis (122), and HLH (64) in patients with DS. The first registered clinical trial for JAK inhibition in DS was completed between 2020 and 2024 using the JAK1/3 inhibitor tofacitinib (Xeljanz), with a focus on safety, improvement of immune skin pathology, normalization of immune markers, and exploratory investigation of effects neurodevelopmental outcomes (NCT04246372). An interim analysis of the first 10 participants showed marked improvements in alopecia areata, psoriasis, and psoriatic arthritis, alongside normalization of IFN scores, cytokine levels, and circulating autoantibodies (25). JAK inhibition was well tolerated with no major immunosuppressive complications over the study period. Participants in this first trial who had a history of DSRD experienced improvement in cognitive, behavioral, and functional domains when taking tofacitinib (123), supporting the launch of a second clinical comparing the JAK inhibitor to intravenous immunoglobulin (IVIG) and the benzodiazepine lorazepam (NCT05662228), which is expected to complete activities in 2026. A third clinical trial testing JAK1-specific inhibitors for the treatment of immunodermatological conditions has been announced. A fourth clinical trial open to anyone with DS ages 6–22 without requirement of a co-occurring condition is currently open to recruitment (NCT07598643). Altogether, current evidence positions JAK inhibitors as the most mechanistically targeted therapy to counter IFN-driven pathology in DS.

JAK inhibitors should be considered for all patients with DS who have an underlying medical condition requiring treatment where JAK inhibitors are approved therapies by the United States Food and Drug Administration or comparable agencies outside of the United States. Dependent on the region, approved indications included (but are not limited to): arthritis (multiple forms), ankylosing spondylitis, ulcerative colitis, atopic dermatitis, psoriasis, vitiligo, alopecia areata, giant cell arteritis, and myelofibrosis. Off-label usage for other autoimmune diseases or immune disorders such as HLH could be considered. IFN scores or cytokine panels could also help guide patient selection, but more data are still needed on patient stratification for use of JAK inhibitors in DS.

### IVIG in DSRD

IVIG has become a core therapeutic option for DSRD, where emerging data support an immune-mediated pathophysiology (124). Multicenter retrospective analysis of individuals with DSRD demonstrated that IVIG is associated with >80% of treated patients showing partial or complete recovery of language, behavior, sleep regulation, and activities of daily living (125). Responses were most pronounced in individuals with neurodiagnostic abnormalities, including cerebrospinal fluid (CSF) pleocytosis, elevated IgG index, and oligoclonal bands; but IVIG effectiveness remained high even among those with normal studies, suggesting a broader therapeutic mechanism (125). Improvement with IVIG in seronegative individuals further indicates that benefit is not limited to antibody-mediated autoimmunity (126). Despite its effectiveness, IVIG responses may wane with discontinuation, and many patients require maintenance infusions or transition to steroid-sparing immunomodulators for sustained stability (126). Even when not all DSRD cases respond to IVIG treatment, current evidence positions IVIG as a well-tolerated and clinically impactful therapy that addresses key immunologic disturbances implicated in DSRD.

### B cell–depleting therapies in DS

B cell dysregulation is a consistent immunologic feature of DS, including expansion of CD11c^+^ T-bet^+^ ABCs and increased production of autoreactive and polyreactive antibodies (34). These abnormalities provide a plausible rationale for selective B cell–targeting in DS-associated autoimmune and neuroimmunological conditions. In DSRD, anti-CD20 rituximab therapy has been used in individuals with clinical or neurodiagnostic features of autoimmune encephalitis (125). However, multicenter analysis indicates that responses to rituximab are variable and generally less robust than those observed with IVIG (125). Benefit appears limited to a subset of patients with demonstrable immune activation on CSF or imaging studies or to those who initially responded to other immunomodulatory therapies before exhibiting relapse (125). Case literature also documents significant clinical improvement in seronegative DSRD using non-B cell–directed immunotherapies, underscoring that B cell pathology is contributory but not universally primary in disease expression (126). Collectively, available data suggest that B cell depletion may be an appropriate adjunctive therapy in carefully selected DS patients with evidence of antibody-mediated or B cell–driven autoimmunity, but current evidence supports a selective, rather than routine, role for anti-CD20 strategies in DSRD and related immune manifestations.

### Corticosteroids in DS

Corticosteroids are commonly employed across immune-mediated and hyperinflammatory conditions in DS, though their role is predominantly as a rapid-acting immunosuppressive rather than a disease-modifying therapy. In DS-associated HLH, high-dose systemic corticosteroids remain an essential component of initial immunosuppression and are routinely administered during acute hypercytokinemic episodes (64). Case reports of relapsing HLH in young children with DS describe repeated courses of pulsed methylprednisolone to achieve rapid control of macrophage activation before escalating to IFN antagonists or JAK inhibitors (64). In DSRD, empiric trials of corticosteroids have been implemented based on suspected neuroimmune activation (126). Clinical observations document partial and often transient improvement in catatonia, sleep disruption, and cognitive slowing, with recurring symptoms during dose reductions, indicating incomplete suppression of underlying pathology (126). Many patients subsequently transition to steroid-sparing agents to maintain stability (126). In DS-associated arthritis, corticosteroids may be used as short bridging therapy (127). Overall, corticosteroids provide effective short-term control of immune activation in DS but rarely offer durable remission as monotherapy.

### Other biologic therapies in DS

A range of biologic therapies has been applied to immune dysregulation in DS, reflecting the broad cytokine abnormalities associated with IFN-driven inflammation. TNF inhibitors such as etanercept and infliximab have been used in DS-associated arthritis, with clinical responses similar to those observed in idiopathic autoimmune disease, although based primarily on clinical experience rather than comparative clinical trials (127). IL-6 blockade with tocilizumab has been employed in refractory DS-associated arthritis and in selected hyperinflammatory presentations to suppress IL-6-mediated acute phase responses (127). IL-1 inhibitors, including anakinra, have been trialed in DS patients with severe hypercytokinemia, particularly those with virus-triggered hyperinflammation or evolving macrophage activation, though clinical responses have been variable (64). More targeted IFN pathway modulation is supported by compelling DS-specific data. In two infants with T21 and relapsing HLH, IFN-γ neutralization with emapalumab induced disease control, and subsequent maintenance with baricitinib achieved sustained remission where standard therapies had failed (64). Mechanistic studies demonstrating that IFNR triplication drives immune hypersensitivity in DS suggest that additional IFN-targeted strategies, such as IFNR blockade, may hold future therapeutic potential (37). Together, these observations highlight an expanding role for cytokine- and pathway-specific biologics in DS immune dysregulation.

## Discussion

**Figure 4.**
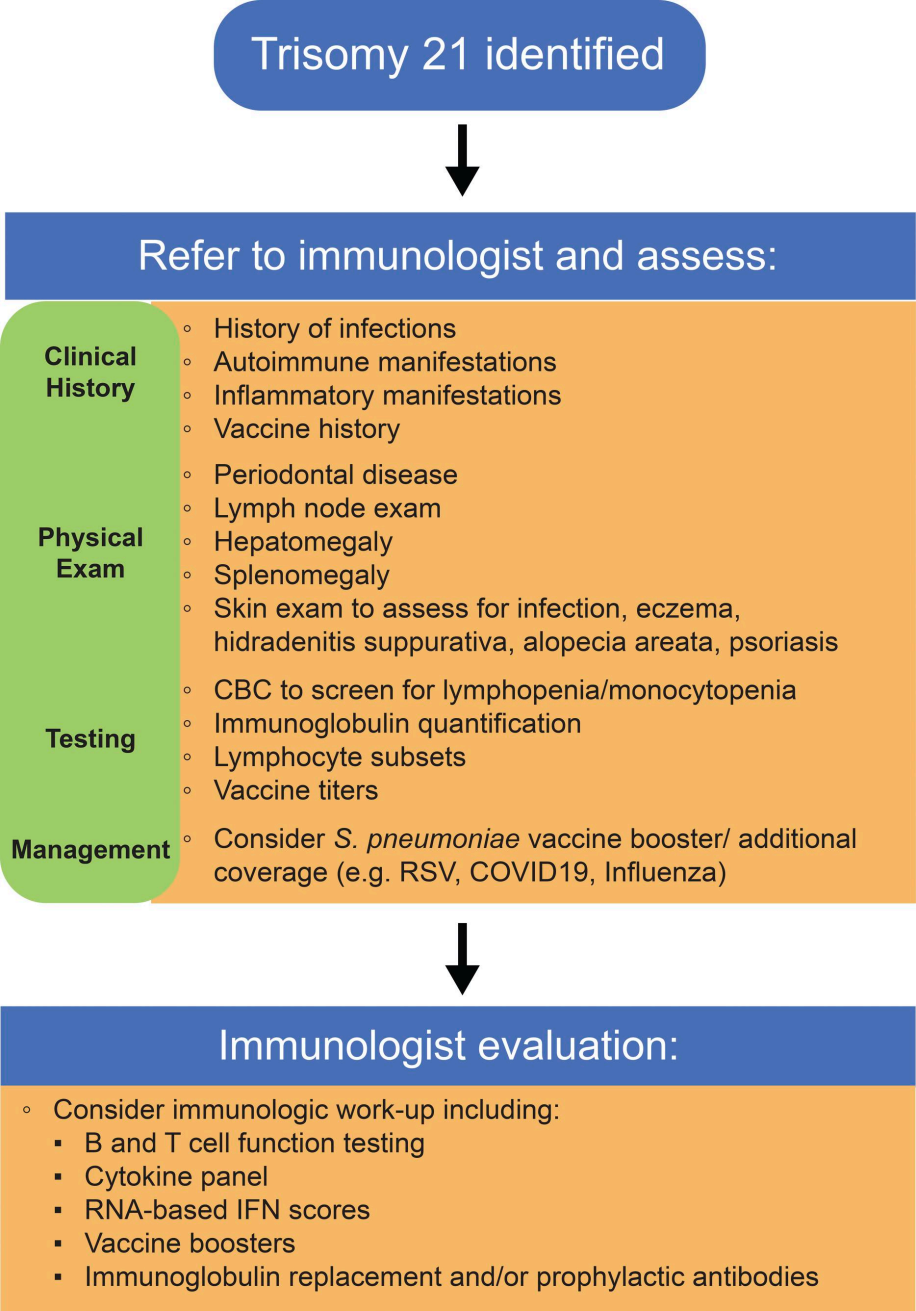
**After diagnosis of DS, referral to an immunologist is recommended.** Immunologists should obtain a clinical history and physical exam focused on the unique immunodeficiency and immune dysregulation findings common in individuals with DS. Initial testing can start with complete blood cell count with differential, immunoglobulin quantification, lymphocyte subsets, and vaccine titers. Management should start with optimizing vaccine responses through boosters if needed and further immunological evaluation and treatment can be considered.

During monitoring of newborns with DS, there is a great emphasis in clinical guidelines on prompt evaluations by cardiology and endocrinology due to the obvious high risk of early mortality from untreated pathology (3). However, with advances in care, patients with DS are living longer and are at greater risk for mortality from infection and immune dysregulation, which requires prompt screening, recognition, and treatment (Fig. 4). While there are distinct aspects of immunity unique to DS, many of the immunological changes seen in DS are also common in older adults and are consistent with a premature senescence of the immune system in DS (128, 129). This premature senescence and pro-inflammatory state likely contribute to the overall immune dysregulation observed in DS and is likely a key factor limiting further gains in life expectancy.

Much more research is still needed in DS (130, 131, 132). It is necessary to determine phenotype and endotype in DS patients with regards to immunodeficiency, autoimmunity, immune dysregulation, and other clinical characteristics. This may help anticipate the need for medical intervention prior to the development of clinical manifestations (Fig. 4).

Although DS does not easily fit the criteria employed by the IUIS for inborn errors of immunity—namely a damaging germline variant in single gene (133)—we propose that DS should be added to IUIS list of inborn errors of immunity, as it is clearly a genetic disorder with many aspects of immune dysregulation and immunodeficiency. However, regardless of its inclusion by the IUIS, it is imperative for clinicians caring for persons with DS to recognize DS as an immunocompromising condition that requires screening, monitoring, and early intervention.

Furthermore, we suspect that the immune system plays a significant role in many other phenotypic characteristics of DS that are not traditionally attributed to the immune system but that so far do not have other reasonable explanations. For instance, the etiology of stunted growth, cognitive differences, and co-occurring neurological and psychiatric issues in DS is not known. While there is a dearth of evidence to support a causation at this time, a potential role for immune dysregulation should be discussed. With this in mind, early and perhaps lifelong immune modulation could be considered to manage and prevent complications often observed in DS, but more research is still needed on this topic (25).

Lastly, it is clear that individuals with DS can greatly benefit from specialized care and evaluation by an immunologist regularly if available to the patient (Fig. 4). We do believe that general practitioners can screen for immunodeficiency in DS with basic commercially available immunological testing such as complete blood cell count with differential, lymphocyte subset quantification, immunoglobulin quantification, and vaccine titers. We recommend for those patients with DS diagnosed in infancy, an initial evaluation by an immunologist around 6–12 mo of age is ideal. This is typically prior to patients with DS receiving live injectable vaccines, though it is rare for a patient with DS to be severely lymphopenic and not be able to receive live vaccines. This is also after infants have completed some of their primary vaccine series and vaccine titers can be checked for efficacy. For patients with a normal evaluation, they should be re-evaluated every few years by an immunologist or sooner if severe or recurrent infections. However, despite the abundance of scientific and clinical evidence detailed in this manuscript, current health supervision guidelines for children and adults with DS do not yet provide any specific recommendations for immune evaluation or surveillance (3, 20).

## Conclusion

The authors believe that this publication should be utilized in clinical practice by immunologists and referring providers to perform regular assessment of immune function as standard of care in patients with DS to reduce morbidity and mortality in this vulnerable population.
